# The EAR Motif in the *Arabidopsis* MADS Transcription Factor AGAMOUS-Like 15 Is Not Necessary to Promote Somatic Embryogenesis

**DOI:** 10.3390/plants10040758

**Published:** 2021-04-13

**Authors:** Sanjay Joshi, Christian Keller, Sharyn E. Perry

**Affiliations:** Department of Plant and Soil Sciences, University of Kentucky, Plant Science Building, Lexington, KY 40502, USA; sanjay.joshi@uky.edu (S.J.); ckeller172@gmail.com (C.K.)

**Keywords:** AGL15, MADS-box gene, *Arabidopsis thaliana*, EAR motif, repression, somatic embryogenesis, seed

## Abstract

AGAMOUS-like 15 (AGL15) is a member of the MADS domain family of transcription factors (TFs) that can directly induce and repress target gene expression, and for which promotion of somatic embryogenesis (SE) is positively correlated with accumulation. An ethylene-responsive element binding factor-associated amphiphilic repression (EAR) motif of form LxLxL within the carboxyl-terminal domain of AGL15 was shown to be involved in repression of gene expression. Here, we examine whether AGL15′s ability to repress gene expression is needed to promote SE. While a form of AGL15 where the LxLxL is changed to AxAxA can still promote SE, another form with a strong transcriptional activator at the carboxy-terminal end, does not promote SE and, in fact, is detrimental to SE development. Select target genes were examined for response to the different forms of AGL15.

## 1. Introduction

AGAMOUS-LIKE 15 (AGL15) is a MADS-domain transcription factor (TF) that accumulates to the highest levels during embryogenesis [1,2,3]. Previous studies have shown AGL15 accumulation promotes somatic embryogenesis (SE), including from the shoot apical meristem (SAM) [4,5]. In the shoot apical meristem somatic embryo (SAM SE) system, mature seeds complete germination and grow in culture media with the synthetic auxin 2,4-D [6]. By three weeks of culture, seedlings will have callused cotyledons and a fraction of the seedlings will have somatic embryo development at the shoot apex region. SE is a valuable model for understanding zygotic processes that occur embedded in maternal tissues. In addition, SE is a mode of plant regeneration and is important for both basic and applied research. However, how cells reprogram to form SE is not well understood (for a recent review [7]). Prior work has documented direct and indirect targets of AGL15, revealing that AGL15 can act as both an inducer and repressor of gene expression and that it controls genes relevant for SE and seed development [8].

To address how AGL15 may induce some direct targets but repress other direct targets, a yeast 2-hybrid screen was performed with the expectation that protein-AGL15 interactions may impact AGL15’s function at different loci. One protein identified in this screen was SAP18 (*Arabidopsis thaliana* SIN3 associated protein P18, At2g45640) that is part of a SWI-INDEPENDENT/HISTONE DEACETYLASE (SIN3/HDAC) complex. HDACs remove acetyl groups from histones, resulting in more compact chromatin and typically a decrease in gene expression of associated genes (for review, see [9]). Specifically, HDA19 and HDA6, two histone deacetylases (HDA) of the RPD3/HDA1 family, interact with SAP18, and HDA19 can interact weakly with AGL15 directly in yeast [10,11]. An ethylene-responsive element binding factor-associated amphiphilic repression (EAR) motif within the C-terminal domain of AGL15 is necessary for interaction with AtSAP18. When this motif, which has the form of LxLxL, has the leucines changed to alanines (AxAxA), SAP18 no longer interacts with AGL15 [10]. AGL15 has also been found to interact with TOPLESS (TPL) and TOPLESS-RELATED PROTEIN2 (TPR2) that also recruit HDA19 [12]. Likewise, EAR repression motifs are involved in the interaction with TPL/TPRs [12].

Several transcription factors key for embryogenesis were found to be AGL15-direct up-regulated targets including *LEAFY COTYLEDON2 (LEC2)*, *FUSCA3 (FUS3)*, and *ABSCISIC ACID INSENSITIVE3 (ABI3)* [8]. Because *LEC2* is well known to enhance SE when overexpressed, perhaps AGL15′s ability to enhance SE is mainly (or entirely) due to its ability to induce gene expression, including that of *LEC2*. Thus, we asked if we changed AGL15 such that its repressive ability is lost and it only acts as an inducer of gene expression, would it still be able to promote SAM SE? We made two modified forms of AGL15 and report here that a form where the leucines within the EAR domain are changed to alanines can still promote SAM SE, but a form that includes a strong transcriptional activation domain reduces the ability to form SAM SE.

## 2. Results

### 2.1. AGL15 Lacking an EAR Domain Promotes SAM SE, but Addition of a VP16 Domain Inhibits SAM SE

**Figure 1:**
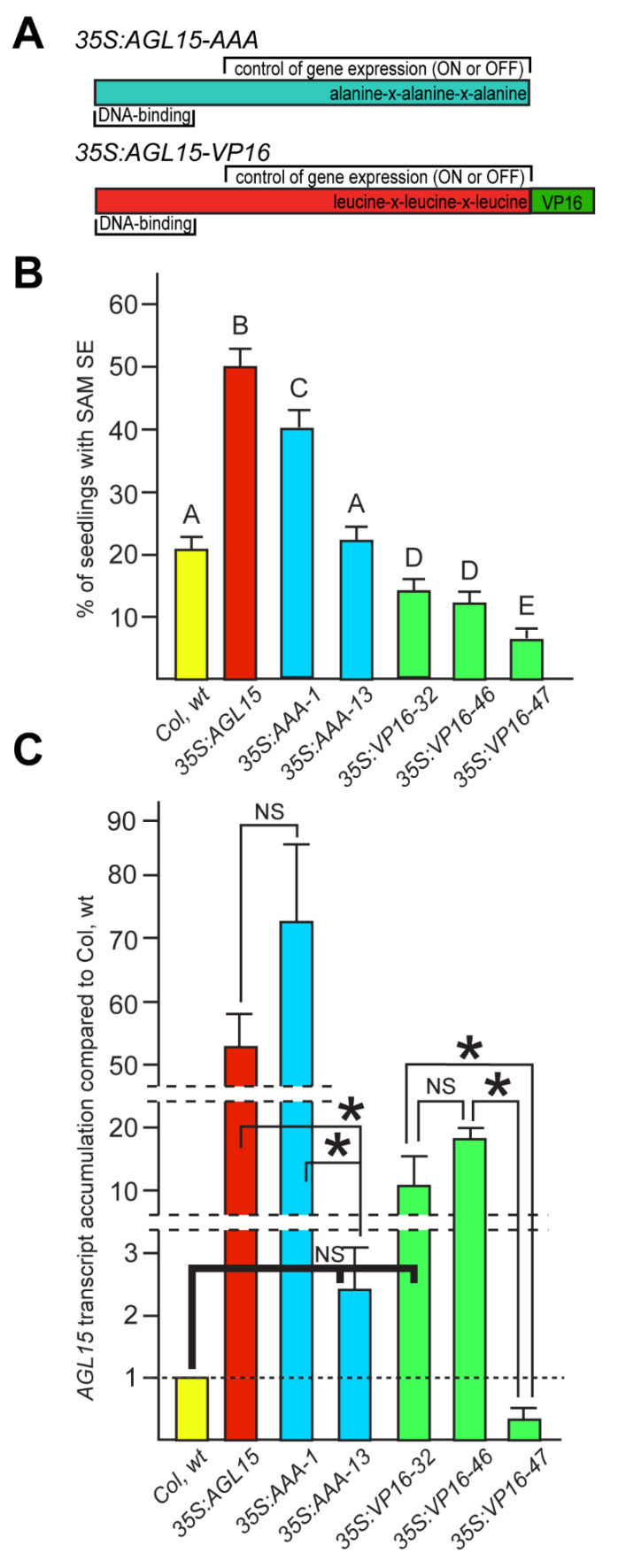
Shoot apical meristem somatic embryo (SAM SE) and transcript accumulation from different AGL15 transgenes. (**A**) Forms of the *35:AGL15* transgene predicted to remove AGAMOUS-like 15 (AGL15)’s repressive activity are shown. In the *35S:AGL15-AAA (35S:AAA)*, the codons for the leucine in the ethylene-responsive element binding factor-associated amphiphilic repression (EAR) motif are altered to encode alanines. In the *35S:AGL15-VP16 (35S:VP16*) transgene, sequences encoding a strong transcriptional activation domain, VP16, are fused at the carboxyl-terminal end of AGL15. (**B**) SAM SE production by the different *AGL15* transgenes compared to Col wild type (wt). Means and standard error of the mean for at least four biological replicates of the experiment are shown. Different letters indicate significant difference in SAM SE production between the different genotypes at *p* < 0.05 using a Student’s *t*-test. (**C**) *AGL15* transcript accumulation in the different transgenic lines compared to Col wt (set to 1) in 10–12 d SAM SE tissue. Data shown are the means of at least two biological replicates of the experiment. * indicates a significant difference at *p* < 0.05. Only nonsignificant (NS) changes are noted for the transgenic lines compared to Col wt. Please note the breaks in the y-axis.

Two different forms of AGL15 that are predicted to eliminate the repressive function were generated (Figure 1A). In one, we changed the EAR domain (LxLxL) such that the leucines became alanines (AxAxA, referred to as *35S:AGL15-AAA*). This would be predicted to eliminate interaction with SAP18 and TPL/TPR, and recruitment of HDACs through these proteins [10,11,12]. The other modification involved fusing regions encoding a VP16 transcriptional activation domain onto AGL15, which may override the repressive activity of AGL15 [13]. Although it has been reported that EAR domains can override the VP16 domain [14], this form of AGL15, referred to as AGL15-VP16, was able to activate a reporter construct in planta [10].

Transgenic lines were assessed for ability to produce SAM SE and for *AGL15* transcript accumulation. As shown in Figure 1B, *35S:AGL15-AAA* was able to promote SAM SE when transcript accumulated to higher levels (line 1, Figure 1C). Lines with lower accumulation of *AGL15-AAA* transcript (*35S:AGL15-AAA-line 13*) did not show a significant increase in SAM SE over all biological replicates (Figure 1B,C). However, individual experiments did show a significant increase in SAM SE (not shown), possibly reflecting the moderately higher transcript accumulation in this line (Figure 1C). SAM SE levels (40%) were significantly higher in *35S:AGL15-AAA-1* compared to wild type (wt). This was the highest expressing line we obtained and transcript accumulation was comparable to *35S:AGL15*. The form of AGL15 with the VP16 domain produced significantly reduced SAM SE (Figure 1B), even with lower levels of transcript accumulation than *35S:AGL15* (Figure 1C). Interestingly, *35S:AGL15-VP16-line 47* shows significantly reduced *AGL15* transcript accumulation compared to Col wt. Not only is the *35S* promoter not driving expression, but because this transgene is in the Col wt background, and there is a significant reduction of *AGL15* transcript compared to Col wt (the primers will amplify both transcript from the endogenous gene as well as the transgene), there appears to be cosuppression. As expected for reduced *AGL15* expression, there is a significant decrease in SAM SE for this line.

### 2.2. 35S:AGL15-AAA Can Decrease Accumulation of AGL15 Transcript from the Endogenous Gene

**Figure 2:**
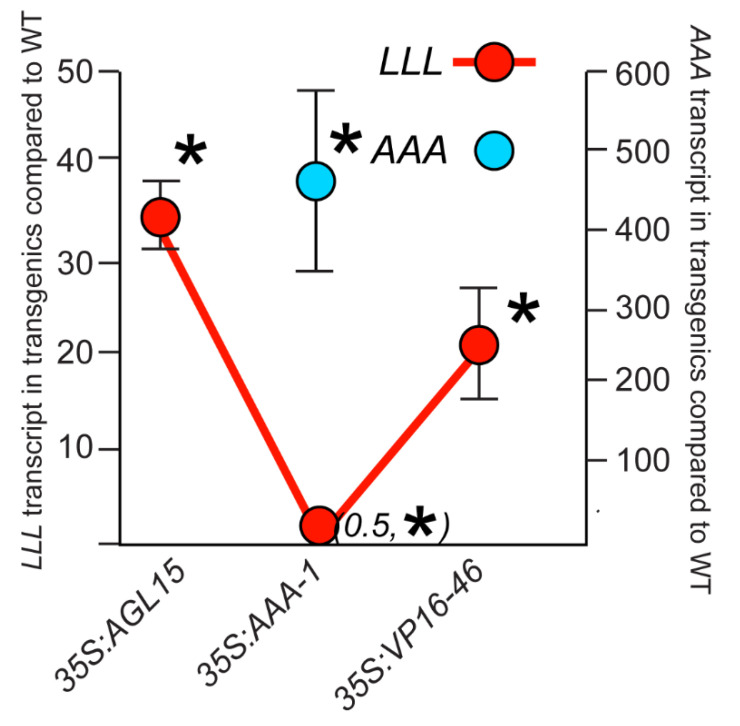
Transcript accumulation from the AGL15 transgenes and the endogenous AGL15 gene. Accumulation of *AGL15* transcript in the transgenics is compared to Col wt (set to 1). The primers that amplify the LxLxL form will hybridize to transcript from the endogenous gene as well as transcript from the *35S:AGL15* and *35S:AGL15-VP16* transgenes. The primer that hybridizes specifically to the AxAxA form does not amplify the LxLxL form. Oligonucleotide primers used are presented in Table S1. Results are means and standard error of the mean from four biological replicates assessing transcript accumulation in 10–12 d SAM SE tissue. * indicates a significant difference at *p* < 0.05 as assessed using a Student’s *t*-test. Numbers in parentheses indicate fold change compared to Col wt for comparisons difficult to see on the scale shown. ns, not significant.

We previously reported that AGL15 represses its own expression [15]. Therefore, one possible explanation for why the AAA form of AGL15 could still promote SAM SE was that this form no longer repressed the endogenous *AGL15* gene expression. Potentially there would be increased *AGL15* transcript from the endogenous gene causing increased SAM SE. To examine this, we generated oligonucleotides that could differentiate between transcript from the endogenous gene (LxLxL) and transcript from the *35S:AGL15-AAA1* transgene (AxAxA oligonucleotide) (Table S1). As shown in Figure 2, the *35S:AGL15-AAA1* tissue accumulated high levels of transcript from the transgene, as expected, but actually showed a significant reduction of transcript from the endogenous gene (the LxLxL form). Thus AGL15-AAA is not promoting SAM SE by upregulation of the endogenous gene; in fact, this form of AGL15 is still able to repress expression from the endogenous gene.

### 2.3. Can All of the Transgenic Forms of AGL15 Promote Expression of LAFL Genes?

**Figure 3:**
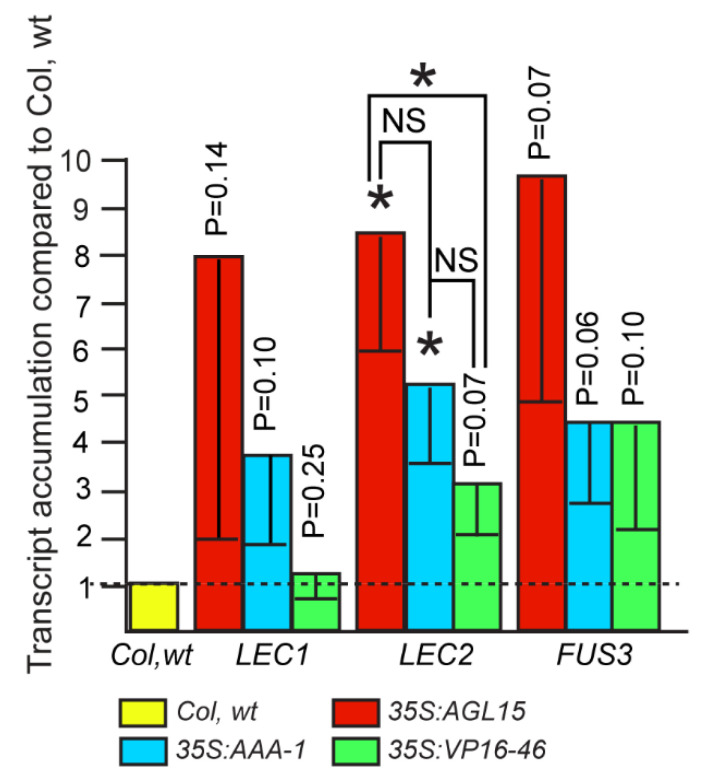
Transcript accumulation from genes encoding key embryo transcription factors in response to the different forms of AGL15. The results shown are the means and standard error of the mean for at least five biological replicates. * indicates significance compared to Col wt or the indicated comparison at *p* < 0.05 as assessed with a Student’s *t*-test. NS, not significant. Differences in transcript accumulation for *LEC1* and *FUS3* between the different transgenic backgrounds were NS for all biological replicates considered as a whole.

One hypothesis about how *AGL15* overexpression promotes SE, including SAM SE, is that it may up-regulate genes such as *LEC2*, *FUS3* and *LEAFY COTYLEDON1* (*LEC1)* that have been shown to promote SE or SE programs post-embryonically [16,17,18,19,20]. While *ABI3* is a direct target of AGL15 [8], it is more restricted in embryonic programs it can drive ectopically [21]. These four genes are often referred to as *LAFL*. If the promotion of expression of these key embryo TFs was all that was needed for SE, and repression of other genes did not factor into ability to promote SE, we would expect the *35S:AGL15-VP16* lines to have abundant SE development because *LEC2* and *FUS3* are direct targets, albeit *LEC2* is a weaker target with a peak at the 3′ end of the gene that is below cutoffs used in CisGenome to call peaks (Figure S1). We would expect that with the strong transcriptional activation domain, this form of AGL15 can still upregulate important controllers of embryogenesis. However, *35S:AGL15-VP16* lines show less SAM SE, even when transcript accumulation is at lower levels than our control for promotion of SE development, *35S:AGL15.* As shown in Figure 3, among all biological replicates, higher level up-regulation of *LEC2* and *FUS3* by *35S:AGL15-VP16* is not occurring, although in both cases transcript is overall higher than Col wt. *LEC1* regulatory regions do not appear to be bound by AGL15 based on the fact that there are intervening genes between the peak and the *LEC1* gene. (Figure S1), and transcript accumulation from this gene appears to be non-responsive to AGL15-VP16. Granted, remote *cis* elements could still be important for regulation of genes that are not direct neighbors of the binding site.

Both *35S:AGL15* and *35S:AGL15-AAA* were able to significantly increase accumulation of *LEC2* transcript, and between these two genotypes for all biological replicates, there was not a significant difference in this measurement. *FUS3* mRNA also accumulated to higher amounts in these backgrounds compared to Col wt, but when considering all biological replicates this was not significantly different from Col wt. However, this is likely due to a wide range of fold increase between replicates: for example, in each of the five biological replicates assessed for *35S:AGL15*, there was a significant increase in *FUS3* mRNA compared to Col wt, but the fold increase varied from 22- to 2-fold.

### 2.4. Other Members of the MADS-Box Family Show Perturbations in Expression in Response to the Different Forms of AGL15

**Figure 4:**
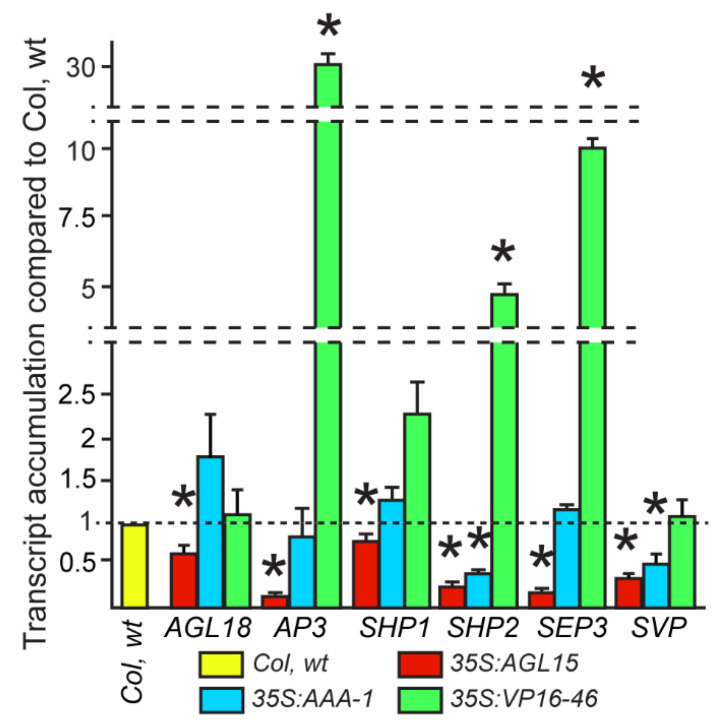
Transcript accumulation from genes encoding select MADS-domain transcription factors in response to the different forms of AGL15. The results shown are the means and standard error of the mean for at least two biological replicates. * indicates significance compared to Col wt at *p* < 0.05 as assessed with a Student’s *t*-test. NS. Please note the breaks in the y-axis. Col wt is set to 1 for all comparisons.

Because *LEC2* and *FUS3*, transcript accumulation is not decreased in *35S:AGL15-VP16* compared to Col wt, but SAM SE is significantly decreased, there appears to be additional factors at play other than regulation of LAFL genes. MADS-domain proteins form higher order complexes with other MADS proteins to drive gene expression and particular complexes may be responsible for different developmental programs (reviewed in [22]). *AGL18* is the closest related paralog to *AGL15* in Arabidopsis and has redundant functions in promotion of SE and in control of flowering time with AGL15 [5,23,24,25]. AGL15 and AGL18 have also been reported to interact via protein–protein interactions [26]. Therefore, we assessed *AGL18* transcript accumulation in response to the different forms of AGL15. Agreeing with a microarray experiment [8] and a preliminary RNA-seq investigation (Joshi and Perry, unpublished), *35S:AGL15* represses transcript accumulation from *AGL18* as confirmed by qRT-PCR compared to Col wt (Figure 4). There is a slight but non-significant increase in this transcript in *35S:AGL15-AAA1*, and no change in *35S:AGL15-VP16-46.* As such, we do not believe increased *AGL18* expression in *35S:AGL15-AAA* is responsible for the ability of this transgene to promote SAM SE.

A number of other MADS-domain proteins besides AGL18 have been reported to interact with AGL15 [27], and many of the genes encoding these interactors are direct targets of AGL15 ([8] and Figure S2). In addition, some of these proteins have EAR motifs and therefore could have redundancy with AGL15 for repressive activity (e.g., SEPALLATA3, SEP3 and SHORT VEGETATIVE PHASE, SVP, in addition to AGL18). Others showed intriguing responses to different forms of AGL15 in the preliminary RNA-seq data (Joshi and Perry, unpublished). Based on these conditions, transcript accumulation of several MADS genes *(APETALA3 [AP3]*, *SHATTERPROOF [SHP]1*, *SHP2*, *SVP* and *SEP3)* were assessed for response to different forms of AGL15 using qRT-PCR. All showed significant reduction in transcript accumulation compared to Col wt when in the *35S:AGL15* background, agreeing with the high-throughput data. We found a significant upregulation of *AP3*, *SHP2* and *SEP3* gene in *35S:VP16-46* line compared to the Col wt control, but not of *AGL18* or *SVP*. *SHP1* did exhibit increased transcript accumulation, but this was not significant. *35S:AGL15-AAA* showed reduced transcript accumulation from *SHP2 and SVP* (Figure 4). Therefore, AGL15-AAA appears to be able to still repress some direct target genes (*AGL15*, Figure 2; *SHP2 and SVP)* but not others (*AGL18*, *SHP1*, *SEP3*, and *AP3*), while AGL15-VP16 can activate some genes (*AP3*, *SHP2* and *SEP3*), but not others (*SVP*, *AGL18*).

## 3. Discussion

Interestingly, elimination of the LxLxL/EAR domain by site directed mutagenesis to replace codons for leucine with codons for alanine (thus generating AxAxA, or as abbreviated AAA) did promote SAM SE when the *35S:AGL15-AAA* transgene was expressed at sufficient levels (i.e., similar to *35S:AGL15*). This modification should eliminate recruitment of proteins needed for histone deacetylation. Histone deacetylation is associated with more compact chromatin leading to repression of gene expression. We had hypothesized that repression of expression of some genes as measured by mRNA accumulation may be important for SE [28], so this was a surprising result. However, we also knew that *35S:AGL15* is able to upregulate *LEC2*, which encodes a gene product that is sufficient to induce SE on seedlings post-germination [19,20]. Thus, it is possible that promotion of SE is solely via a few key factors, such as LEC2, that have been shown to be essential for SE [29]. Indeed, all forms of AGL15 were able as an overall average cause increased accumulation of *LEC2*, and *FUS3* mRNA, and, for LLL and AAA forms, *LEC1*. *FUS3* is a direct expressed AGL15 target and can promote embryo programs after completion of germination, although not to the extent observed for LEC2 and LEC1 [8,16,17]. In contrast to *LEC2* and *FUS3*, *LEC1* is an indirect target, so we would not expect association with AGL15-VP16 and induction of expression, unless this was a result of indirect regulation. While the overall means corresponded with the ability to promote SAM SE, numbers varied between biological replications and results across all experiments were not significant, with the exception of *LEC2* in *35S:AGL15* and *35S:AGL15-AAA*. Thus, this could at least in part explain why AGL15-AAA can promote SAM SE, but does not explain why AGL15-VP16 shows reduced SE as these key regulators are not reduced compared to Col wt in this background.

While other transgenic lines of *35S:AGL15-AAA* besides line 1 showed in some experiments a significant increase in SAM SE, this was not significant over all replicates of the experiment. However, no other lines showed as high an accumulation of *AGL15-AAA* transcript as did line 1, which was comparable to *35S:AGL15*. Thus, it appears that the *AAA* transcript can support SAM SE development when expressed at sufficient levels, but not reproducibly in lines with lower levels of accumulation. No lines of *35S:AGL15-VP16* had levels of transcript accumulation comparable to *35S:AGL15*; line 46 was one of the highest expressing lines. However, even at this level of transcript accumulation, a significant decrease in SAM SE was observed. Similarly, *35S:AGL15-VP16*, line 32 showed a reduction in SAM SE, indicating that strong transcriptional activation of direct targets is detrimental to promotion of SAM SE. Another independent transgenic line (line 47) showed decreased *AGL15* transcript accumulation (Figure 1C). The primers were such that they would detect the transcript from the transgene as well as from the endogenous *AGL15* gene. This line showed a decrease in SAM SE production (Figure 1B), presumably due to reduced *AGL15* transcript accumulation as observed in loss-of-function *agl15* [5].

Because AGL15 has been shown to directly repress its own expression [15], it is possible that the *35S:AGL15-AAA* transgene no longer represses the endogenous *AGL15* gene, leading to up-regulation of the wild type form of *AGL15* (also referred to as LLL to reflect the intact EAR domain). We used primers that were specific to the AAA and the LLL forms and found that the AAA form did not lead to overaccumulation of *AGL15* transcript from the endogenous gene, but rather significantly decreased transcript accumulation compared to wild type (Figure 2). Thus, the LLL motif is not necessary for AGL15 repression of its own expression.

AGL18 has been found to be redundant with AGL15, has an EAR domain, and has been shown to interact with AGL15 [5,23,26]. *AGL18* is also repressed by AGL15, and this is a direct interaction (Figure 4 and Figure S2). The EAR domain is needed for this repression as the AGL15-AAA form no longer represses *AGL18* and in fact there is a slight, but non-significant increase in transcript from this gene.

MADS-domain protein function binds DNA as dimers, either homodimers or heterodimers, but they may also interact as higher order complexes via protein–protein interactions. We examined information available in BioGRID [27] to identify other MADS found to interact with AGL15. Some of these genes encoding interacting proteins that also showed response in a preliminary RNA-seq experiment were verified by qRT-PCR (Figure 4) and are shown in Table 1. AP3 has not been reported to interact directly with AGL15, but was included because a preliminary RNA-seq experiment showed a large increase in transcript from this gene in response to AGL15-VP16, and both AP3, AGL15, and many other MADS do interact with SEP3 that shows a broad pattern of mRNA accumulation and also includes an EAR motif [30,31]. SEP3 has also been shown to include a transcriptional activation domain [32]. SVP is expressed in embryos and also contains an EAR domain. We tested the forms of AGL15 and while LLL repressed these genes, VP16 showed significant over-accumulation of mRNA for three of them (Figure 4). Interestingly, these three genes are not associated with development in embryo mode as summarized in Table 1. Two of these genes are not expressed in an embryonic culture tissue (ECT) [33]. This ECT is a SE tissue derived from *35S:AGL15* zygotic embryo explants placed on MS medium without exogenous hormones. The ability to produce secondary embryos was positivity correlated with AGL15 accumulation [4]. The secondary embryos could be subcultured leading to long-term maintenance of the tissue developing in embryo mode (the oldest cultures are nearly 25 years old; S. Perry, unpublished observation). Furthermore, transcript accumulation patterns from public databases [34] were examined to assess which of these genes are normally expressed in zygotic embryos (Table 1: data from [35]: TPM: transcript per million >1 is considered expressed). None of the three MADS-box genes significantly up-regulated by AGL15-VP16 are expressed in zygotic embryos (TPM < 1). However, *SHP1* that is associated with ECT and zygotic embryo development is responsive to AGL15-VP16.

The ability of the SAM in the SAM SE system to produce SE can be interpreted in several ways. The initial observation of SAM SE production involved mutants with unusually large meristems and induction of SE with 2,4-D could promote SE from these meristems [6]. However, *35S:AGL15* was found to increase SAM SE without at least an initially larger meristem [4]. Because one of the few contexts that AGL15 can be detected by immunolocalization after completion of germination is the very young seedling SAM (4 d seedlings) that is reduced by 6 d, an interpretation of *35S:AGL15* increasing SAM SE was that the SAM remains “embryo” longer due to continued AGL15 accumulation in nuclei of cells. This, along with 2,4-D, could allow “expression” of this embryo context. Although *AGL15* transcript is very low in the SAM, auxin, as used in SE systems, up-regulates *AGL15* [15], and overexpression of *AGL15* (and *AGL18*) causes delays in the transition to reproductive development. In addition, the *agl15 agl18* mutant flowers early in short days [23,24]. SVP and AGL24 are also involved in this transition [25]. While *AGL24* did not show significant responses to the different forms of AGL15, *SVP* did. Therefore, we were curious as to the trend for the other MADS investigated. As shown in Table 1, *AGL18* and *SVP* show decreases in transcript accumulation as the SAM transitions through the plant life cycle. Meanwhile *AP3* and *SEP3* increase in transcript abundance as the SAM progresses through the plant life cycle.

Finally, AGL15 has been proposed to influence dedifferentiation, perhaps by regulating stress responsive genes including transcription factors associated with dedifferentiation [36,37]. Therefore, we also looked at trends in transcript accumulation in datasets involving culture of explants on callus-inducing medium (Table 1).

**Figure 5:**
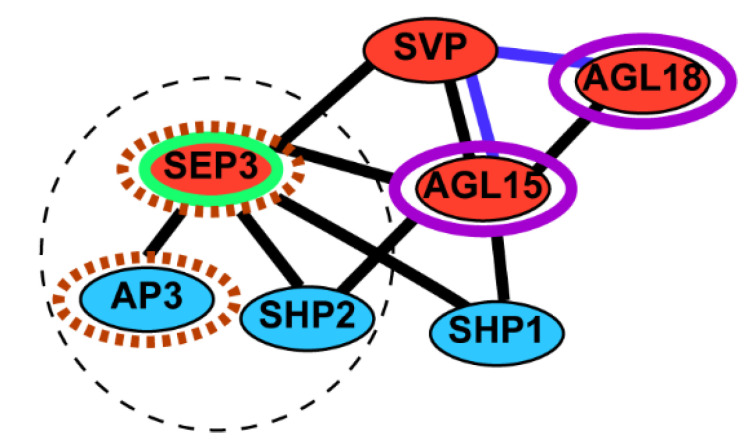
Interactions among MADS-proteins in this study. Lines connecting MADS indicate evidence of physical (black) and/or genetic (blue) interactions as complied in BioGRID. Red indicates the presence of and EAR/LxLxL motif in the protein. The green outline on SEP3 indicates evidence for transactivation activity [32]. The purple outline highlights MADS associated with embryo development, decreased transcript accumulation with the transition to reproductive development and increased transcript accumulation with dedifferentiation on callus-inducing medium as summarized in Table 1. Brown dotted outlines indicate the opposite pattern. The black dotted circle highlights the subset of genes studied that respond to AGL15-VP16.

Summarizing these observations, it is interesting that the MADS-box genes that respond to AGL15-VP16 tend to 1, not have detectable mRNA in a somatic embryo culture tissue [33] and/or low-to-no transcript in zygotic embryos [35], 2, increase in transcript accumulation in the apical meristem as it transitions from vegetative to reproductive [38] and 3, tend to be downregulated when dedifferentiation is induced on callus-inducing medium [39,40]. Conversely, those non-responsive show the opposite patterns of transcript accumulation (Table 1). Although AP3 forms dimers with PISTILLATA (PI) to bind DNA [41,42], *PI* is expressed in embryo contexts [33,35]. Even if AP3 (and possibly proteins encoded by other ectopically expressed genes) does not form dimers that can bind DNA, it can still interact with other MADS and prevent formation of the complexes needed to drive particular developmental programs. Not all MADS-domain proteins of the MIKC class can tetramerize to form higher order complexes, and in this regard, it may be relevant that SEP3 is responsive to AGL15-VP16 because it can form tetramers [43] and has in fact been described as the “glue” involved in many MADS protein complexes [30]. Thus, as summarized in Figure 5, one model for why AGL15-VP16 inhibits SAM SE is that the ectopic expression of MADS that are not associated with embryo context and/or differentiation disrupts complex formation to control gene programs to promote SE. Further work to assess transcriptomes will be revealing.

Why are some genes (e.g., *AGL15*, *SVP*, *SHP2*) still able to be repressed by the AAA form but others (*AGL18*, *AP3*, *SHP1 and SEP3*) are not? All of these targets are potentially directly regulated ([8] and Figure S2), and all are responsive to *35S:AGL15*. It is possible that AGL15 forms different complexes at different sites to control targets, and in some cases, products of other genes such as AGL18 and/or SEP3 can provide a repressive function. There are also passive and active models of gene repression where passive repression does not recruit chromatin modifying complexes to turn off gene expression but rather displaces or competes with another factor that would normally activate the gene. Active repression would be expected to involve chromatin modifications. Further work looking at chromatin states at various loci should be instructive. In addition, AGL15 was able to interact directly with HDA19, although weakly in yeast-2 hybrid assays [10] and perhaps this interaction does not involve the EAR domain.

Additionally, why can AGL15-VP16 activate some direct targets (*AP3*, *SEP3*, and *SHP2*) but not others (*AGL18* and *SVP)*? More work needs to be performed, but again, perhaps particular interactions at different loci may lead to these different responses. While addition of VP16 clearly activates some targets, presumably by overriding repressive domains, others have reported that the EAR domain within other proteins can override the transcriptional activation of the VP16 domain. It is intriguing that the genes that are activated by AGL15-VP16 appear to have one binding site associated, whereas *AGL18* shows association with AGL15 at the 5′ and the 3′ ends of the gene, while *SVP* has multiple regions at the 5′ end of the gene associated with AGL15 (Figure S2). Further investigation is needed to see if this is generally true.

## 4. Materials and Methods

### 4.1. Generation of Transgenic Lines

Construction of *35S:AGL15* and *35S:AGL15-VP16*, and site directed mutagenesis of the codons encoding the EAR domain to AxAxA are described in [10,24]. All constructs were introduced by the floral dip method [44] into Col. Transgenic lines were selected by Kanamycin resistance. All lines used in subsequent analyses were single insert, hemizygous lines. This replicated the situation for *35S:AGL15* where homozygous transgenic lines were sterile and the plants must be propagated as hemizygotes.

### 4.2. SAM SE

Seeds of Columbia wild type (Col wt) and the transgenic lines were surface sterilized as done by [4]. Seeds were chilled for 2 to 3 days at 4 °C and introduced into SAM SE liquid culture media as described previously [6]. Cultures were incubated on a rotary shaker at 23 °C to 24 °C with a 23-h-light/1-h-dark regime. For RNA extraction, 10–12 days old seedlings from the SAM SE system were collected and flash frozen in liquid nitrogen. No SAM SE development is observed at 10–12 days. After 21 days, the frequency of somatic embryos at the apex of the callused seedlings was determined.

### 4.3. RNA Extraction and qRT-PCR

Total RNA from the frozen samples was isolated using the RNeasy Plant Mini Kit (Qiagen) with 1% polyethylene glycol added to the RLC buffer for seeds/seedlings [45,46]. One microgram of total RNA from SAM SE culture was treated with DNase I (Invitrogen) and used for first-strand cDNA synthesis. cDNA was transcribed using the Reverse transcription system (Promega) following manufacturer’s instructions. For qRT-PCR reactions, an aliquot (0.5 µL) of each cDNA reaction was amplified by specific primer pairs (0.25 microM each; Table S1) in a reaction mix consisting of 1x buffer for Klentaq1 (AB Bioscience LLC, St. Louis, MO, USA), 50 mM KCl, 0.2 mM dNTPs, 1/40,000 diluted SyBr Green (Invitrogen) and 2 units of Klentaq1 in a final volume of 20 µL, using a CFX Connect real-time PCR system (Bio-Rad). Data were processed using CFX Manager software or using REST software [47]. Relative transcript levels were normalized to the expression of the reference gene *TUA3*. Significance was determined using a Student’s *t*-test.

## Figures and Tables

**Table 1:** Expression patterns for MADS-box genes that are responsive to different forms of AGL15 from BAR eFP [34]. Data from analysis of mutants and overexpressors is included for the transition to reproductive development.

Name, AGI	In ECT? [33]	Highest TPM in Embryo; Stage [35]	Trend with Transition to Reproductive SAM [38]	Change with Callus Induction [39,40]
*AGL15*, *At5g13790*	Yes	138; early–mid	Very low; delays [24,25]	Increase (root) [39]
*AGL18*, *At3g57390*	Yes	3.16; early	Decrease; delays [24,25]	Increase (root) [39,40], Decrease (petal), [40]
*AP3*, *At3g54340*	No	0.44; mature green	Increase	Decrease (petal), [40]
*SEP3*, *At1g24260*	Yes	0.34; early	Increase	Decrease (petal), [40]
*SHP1*, *At3g58780*	Yes	57; broad	Very low	Very low
*SHP2*, *At2g42830*	No	0.29; early–mid	Very low	Very low
*SVP*, *At2g22540*	Marginal	13.8; early–mid	Decrease; delays [24,25]	Decrease (petal), [40]

## Data Availability

While no highthroughput data was deposited from this study, we do reference Zheng et al. 2009 [8] and this data is available at NCBI GEO superseries GSE17742.

## Supplementary Materials

The following are available online at https://www.mdpi.com/article/10.3390/plants10040758/s1, Table S1. Oligonucleotides used for qRT-PCR, Figure S1. CisGenome traces showing association of AGL15 with genes encoding key embryo TFs, Figure S2. CisGenome traces showing association of AGL15 with genes encoding other MADS-domain proteins.
