# Supplementary material for: The EAR Motif in the Arabidopsis MADS Transcription Factor AGAMOUS-Like 15 Is Not Necessary to Promote Somatic Embryogenesis
Source: Plants (Basel). 2021 Apr 13;10(4):758. doi: 10.3390/plants10040758 (PMC8069471; doi:10.3390/plants10040758)
Supplement: Supplementary file 1 [file plants-10-00758-s001.pdf]

Table S1. Oligonucleotides used for qRT-PCR. All oligonucleotides are written 5′ to 3′.

AGI	Gene Name	Forward	Reverse
AT5G19770	*TUA3*	TGG TGC CCA ACT GGG TTC AAA T	ACC TCT GCA ACT GCT GTG TTG T
AT5G13790	*AGL15*	TGC AAA GCC TTG AGC AGC AAC T	AAC CTG TCT ACG CAA GGT CTC GTT
	AGL15-LLL	GAC ACA ACT TTG CAA TTA GGG TT	GTT TCG CTG CTC GTG TTT G
	AGL15-AAA	TGC GCA AGC AGG GGC	GTT TCG CTG CTC GTG TTT G
AT1G21970	*LEC1*	AAC GTG GAG CTC CCT TCT CTC ACT AT	TTG GGT TGT TGC TGT GCT GGT
AT1G28300	*LEC2*	ATC CTC AGA TCC CGG TTA CGC AAA	ACC ACC TCT CTC TTG CCA CAA AC
AT3G54340	*AP3*	CAT ACA TGA GCT GGA ACT AAG A	ACG TGA CCC TTC GAT TTG
AT3G58780	*SHP1*	GTA CCT GCG AGC AAA GAT AG	GAA GAT ACA CCG GAT TCG TAA A
AT2G42830	*SHP2*	CGC TCC AAG ATT ACT GAA AGA	CGA CTG GTG AGA AGA AGT AAC
AT1G24260	*SEP3*	TTC GAG CAT GCT TCG GAC ACT	AGG GTC CGA AGC ATG CTC GAA
AT3G57390	*AGL18*	ACC AGT TGA ACG AGA GCT TG	TCT CAA CCT GTT TGC GCA AG
AT3G26790	*FUS3*	TCA TCA TGG GTT ATC GGC GT	AGT TGG CAC GTG GGA AAT AGG A
AT2G22540	*SVP*	ACA GTC GTC GGA GTC TAT T	CTA ACC ACC ATA CGG TAA GC

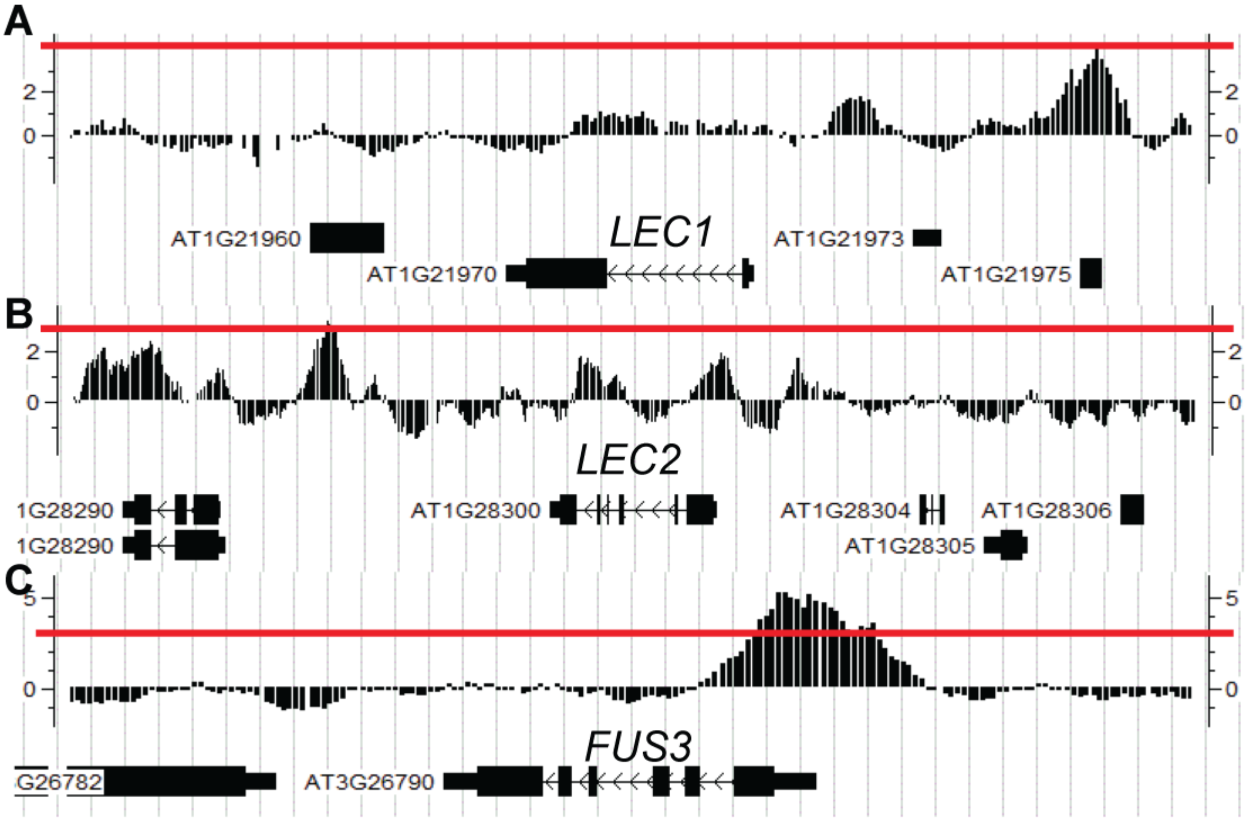

Figure S1: CisGenome traces showing association of AGL15 with promoter regions of (A) *LEAFY COTYLEDON1* (B) *LEAFY COTYLEDON2*, and (C) *FUSCA3*. The red line indicates the moving average cutoff of 3 for peak determination as used in Zheng et al., (2009).

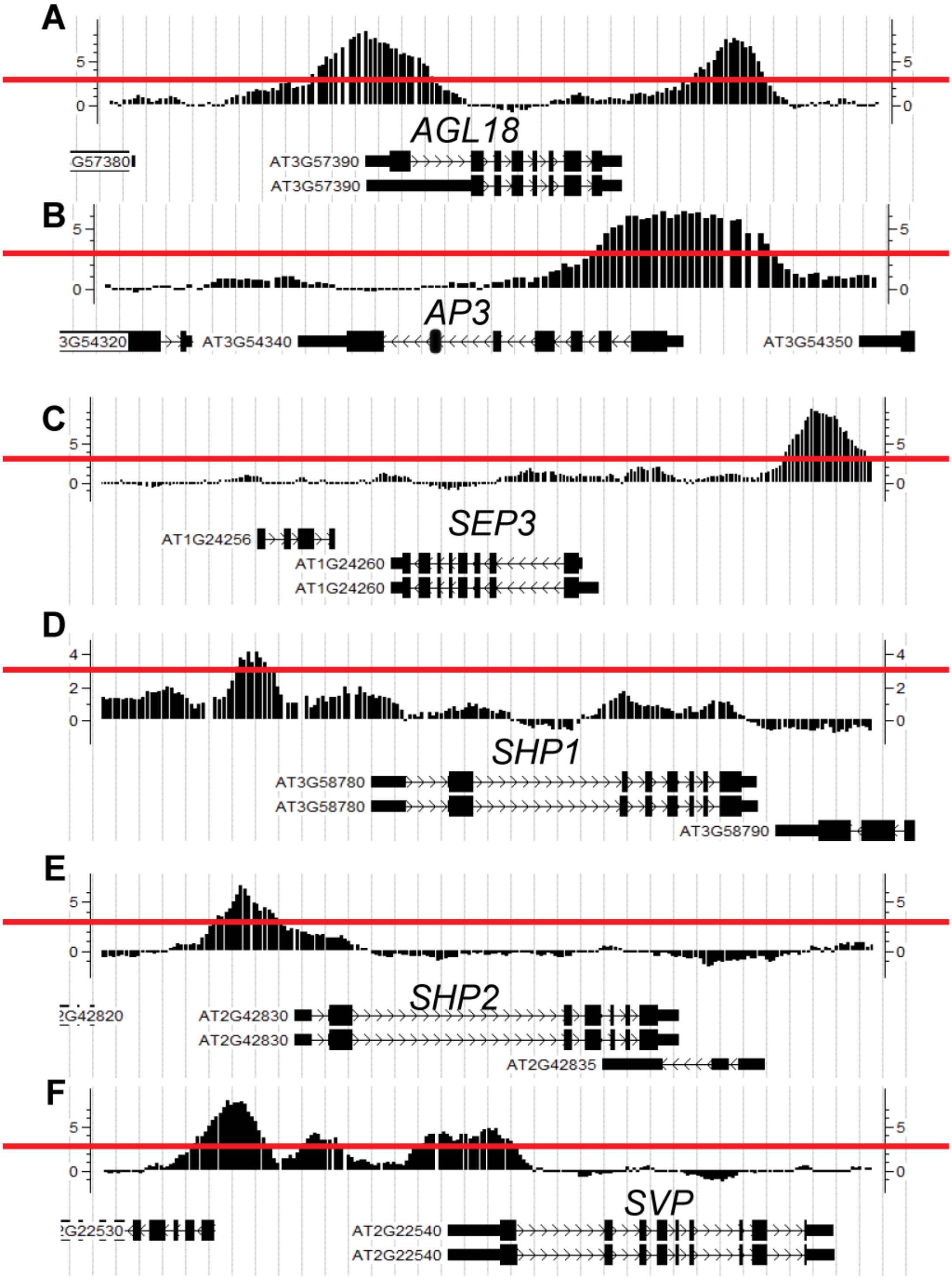

Figure S2: CisGenome traces showing association of AGL15 with promoter regions of (A) *AGAMOUS-Like18* (B) *APETALA3*, (C) *SEPALLATA3*, (D) *SHATTERPROOF1,* (E) *SHAT-TERPROOF2,* and (F) *SHORT VEGETATIVE PHASE*. The red line indicates the moving average cutoff of 3 for peak determination as used in Zheng et al., (2009). In all cases, the binding region(s) is 5' of and/or within the 5' end of the gene, with the exception of *AGL18* that also shows association with AGL15 3' of the gene.
